# A Novel Hydrogen Sulfide Donor Reduces Pilocarpine-Induced Status Epilepticus and Regulates Microglial Inflammatory Profile

**DOI:** 10.3389/fncel.2021.780447

**Published:** 2021-12-02

**Authors:** Zhongrui Liu, Ziting Zhu, Yan He, Qiyun Kang, Fei Li, Wenlong Zhang, Yuehua He, Yuwan Lin, Baoyi Huang, Mingshu Mo, Pingyi Xu, Xiaoqin Zhu

**Affiliations:** ^1^Department of Physiology, School of Basic Medical Sciences, Guangzhou Medical University, Guangzhou, China; ^2^Department of Neurology, Key Laboratory of Neurogenetics and Channelopathies, Institute of Neuroscience, The Second Affiliated Hospital, Guangzhou Medical University, Guangzhou, China; ^3^Department of Neurology, The First Affiliated Hospital, Guangzhou Medical University, Guangzhou, China

**Keywords:** hydrogen sulfide donor, status epilepticus (SE), inflammatory profile, neuroinflammation, pilocarpine

## Abstract

Although epilepsy is one of the most common neurologic disorders, there is still a lack of effective therapeutic drugs for it. Recently, we synthesized a novel hydrogen sulfide (H_2_S) donor, which is found to reduce seizures in animal models effectively. But it remains to be determined for its mechanism. In the present study, we found that the novel H_2_S donor could reduce pilocarpine-induced seizures in mice. It alleviated the epileptic behavior, the hippocampal electroencephalography (EEG) activity of seizures, and the damage of hippocampal neurons in status epilepticus mice. In addition, the novel H_2_S donor could reduce microglial inflammatory response. It not only reduced the upregulation of pro-inflammatory markers [inducible nitric oxide synthase (iNOS) and cyclooxygenase 2 (COX2)] in status epilepticus mice, but also increased the levels of microglial anti-inflammatory marker arginase-1 (Arg-1). In lipopolysaccharide-treated microglia BV2 cells, administration of the H_2_S donor also significantly reduced the lipopolysaccharide-induced upregulation of the expression of the pro-inflammatory markers and increased the expression of the anti-inflammatory markers. Thus, the novel H_2_S donor regulates microglial inflammatory profile in status epilepticus mice and *in vitro*. These results suggested that the novel H_2_S donor can reduce seizures and regulate microglial inflammatory profile, which may be a novel mechanism and potential therapeutic strategy of the H_2_S donor anti-seizures.

## Introduction

Epilepsy, a nervous system disorder characterized by sudden abnormal hypersynchrony of neurons, affects 70 million people in the world. Despite there are various antiepileptic drugs available, approximately 30–40% of patients are refractory to these treatments (Kwan et al., 2010; Thijs et al., 2019). Therefore, it is urgent to explore the pathogenesis of epilepsy and find alternative treatment strategies.

Microglia are brain resident immune cells and are emerging as central players in regulating pathways of central nervous system (Aloisi, 1999). Microglia are phenotypic plastics and can be activated by variety of stimuli to express various inflammatory profile (Hu et al., 2015; Orihuela et al., 2016). In some specific disease states, microglia express more inducible nitric oxide synthase (iNOS) and release more pro-inflammatory cytokine including interleukin (IL)-1β and tumor necrosis factor-α (TNF-α). While under the stimulation of specific drugs or cytokines (such as resveratrol or IL-4), the expression of several proteins including arginase-1 (Arg1) and the production of anti-inflammatory cytokines such as IL-10, IL-4, and IL-13 increased in microglia (Gordon, 2003; Benson et al., 2015; Yang et al., 2017; Therajaran et al., 2020; Zhang et al., 2021). A number of studies have reported that microglial activation was observed in patients and animal models of various types of epilepsy (Vezzani, 2004; Vezzani et al., 2015, 2019). Microglial activation has been recognized as a major contributor to inflammation of the epileptic brain (Vezzani et al., 2015). The “activated” microglia have exhibited heterogeneity in their phenotypes, which makes it difficult to determine whether these microglia are proepileptic or antiepileptic (Hiragi et al., 2018). Both microglial pro-inflammatory cytokines (IL-1β and TNF-α) and anti-inflammatory cytokines (IL-4 and IL-10) showed increased expression after pilocarpine-induced status epilepticus, indicating a complex role of microglia in the epileptic brain (Hu et al., 2015). Microglial pro-inflammatory cytokines have been implicated in epileptogenesis. In contrast, microglial anti-inflammatory cytokines participate in the resolution of the inflammatory processes, thereby potentially limiting epileptogenesis (Gordon, 2003; Therajaran et al., 2020). Therefore, modulation of microglial inflammatory profile will become a potential therapeutic strategy for epilepsy.

Hydrogen sulfide (H_2_S), a traditional toxic gas in the atmosphere, is synthesized endogenously in mammals and recognized as a gaseous signaling molecule that may act as a neurotransmitter in brain (Vandiver and Snyder, 2012; Wang, 2012; Kolluru et al., 2013; Nagpure and Bian, 2015). Concentrations of H_2_S in the brain changes in a variety of neurological diseases including seizures (Han et al., 2005; Bae et al., 2013; Giuliani et al., 2013; Luo et al., 2014; Paul and Snyder, 2014). High concentration of H_2_S has toxic effects, whereas low concentration of H_2_S has neuroprotective effects (Tan et al., 2010; Hu et al., 2011). The downregulation of H_2_S levels results in hippocampal hyperactivity in febrile seizure rats, whereas neural excitability is reduced by administration of NaHS (Han et al., 2005). H_2_S can also prevent damage in the hippocampus caused by recurrent episodes of febrile seizures (Chen et al., 2015). Unfortunately, traditional H_2_S donors including NaHS and other sulfates are easily oxidized to form sulfane sulfursor and cause adverse effects due to quick release (Yang et al., 2020). Recently, we synthesized a novel carbazole-based H_2_S donor, which is safer and has more effective pharmacological administration to release H_2_S (Li et al., 2008; Li Y. F. et al., 2009; Yang et al., 2020; Zhu et al., 2020). We found that the novel H_2_S donor has the effects of neuroprotection and reduction of epileptic seizures (Zhu et al., 2020, 2021; Liu et al., 2021). However, the underlying mechanisms of the H_2_S donor against seizures are poorly understood. In this study, we investigated the potential role of the novel H_2_S donor in regulating microglial inflammatory profile and found that it can decrease microglial pro-inflammatory profile and simultaneously increase the microglial anti-inflammatory profile in pilocarpine-induced status epilepticus mice.

## Materials and Methods

### Animals

Adult male C57BL/6 mice (25 ± 2g, 8–12 weeks old) were purchased from GemPharmatech conditions (ambient temperature: 20 ± 2°C; humidity: 60 ± 5%) with 12 h light/dark cycle, and provided *ad libitum* access to food and water. All mice were randomly divided into four groups: Control group (Ctrl), SE group, H_2_S donor intervention group (SE+H_2_S), and H_2_S donor control group (H_2_S).

Initially, the mice were pretreated with H_2_S donor or dimethyl sulfoxide (DMSO) 2 h before pilocarpine-induced status epilepticus (SE). Then, the behavioral changes and electroencephalography (EEG) of mice were recorded during SE. The mice were sacrificed at various time points (1d, 7d, 14d, 28d) after the SE induction, and the brain was removed and stored at −80°C for corresponding experiments. All the experiments were approved by the Institutional Animal Care and Use Committee of Guangzhou Medical University.

### Pilocarpine-Induced Status Epilepticus Model

After pretreatment with H_2_S donor (500 μM, 5 μl, i.c.v.) or DMSO (5 μl, i.c.v., sigma, United States) for 2 h, animals from both groups (SE and SE+H_2_S) were injected with pilocarpine to induce SE. Specifically, atropine (1 mg/kg, i.p., sigma, United States) was given 30 min prior to pilocarpine hydrochloride (300 mg/kg, i.p. of meilinbio, China) to reduce the peripheral effects. Seizure scores were assessed according to the protocol of a previous study (Mello and Covolan, 1996). Briefly, some mice presented a generalized convulsive (stage 4 or 5) seizure that turned into continuous seizures in the form of limbic motor seizures with intense salivation, rearing, upper extremity clonus, and falling, lasting up to 90–150 min, which characterized SE. Diazepam (10 mg/kg, i.p., King York, China) was injected 90 min after SE onset to inhibit or alleviate SE. The mice that progressed to at least Stage 4 were killed for immunohistochemistry or western blot at various time points.

### H_2_S Donor Pretreatment by Lateral Ventricle Injection

The mice were anesthetized by intraperitoneal injection of 2% sodium pentobarbital, and then fixed on the stereotactic apparatus. The H_2_S donor was delivered at 500 μM in 5 μl of DMSO in mice by i.c.v. injection. The coordinates were as follows: 0.2 mm posterior to bregma, 0.9 mm lateral to the sagittal suture, and 2.0 mm below the subdural surface (Feng et al., 2019; Mo et al., 2019). The needle was remained in place for 10 min and then withdrawn slowly.

### Electroencephalography

Hippocampus EEG was recorded as previously described (Zhu et al., 2021). First, the mice were anesthetized by intraperitoneal injection of 2% sodium pentobarbital (30 mg/kg) and fixed in the stereotactic apparatus. The hippocampus was located as follows: 2.3 mm posterior to bregma, 1.8 mm lateral, 2.0 mm ventral to the duramater. The skull was drilled, and a stainless steel bipolar copper core electrode was inserted into the subdural 3.0 mm. After implantation, all electrostatic electrodes were fixed on the skull with jewel screws and dental acrylic acid. EEGs of mice were recorded by a BL-420E Biological Function Experimental System (Techman, Chengdu, China) for 1 h. Then, the wave amplitudes were measured in microvolts (μV) *via* TM_WAVE version 2.1 (Techman, Chengdu, China) and data were analyzed and counted.

### Western Blotting

The hippocampal tissue or BV2 cells were lysed with radio immunoprecipitation assay (RIPA) lysate (Beyotime, China). The protein concentration was measured by bicinchoninic acid (BCA) Protein Assay Kit (Beyotime, China). Due to the difference in the expression of target proteins (such as IL-10 and Arg-1), the loading mass of total protein was increased up to 80 μg per lane in order to obtain clearer band signals. The total loading volume is controlled within 10 μl per lane to avoid sample overflow. Samples were subjected to 10–12% sodium dodecyl sulfate-polyacrylamide (SDS-PAGE) gel electrophoresis and transferred onto polyvinylidene-difluoride (PVDF, Millipore, United States) membranes. Then, the membranes were blocked with bovine serum albumin (BSA), and incubated with rabbit anti-COX2 (1:500, #12375-1-AP, Proteintech Group, United States), rabbit anti-Arg-1 (1:4,000, #16001-1-AP, Proteintech Group, United States), rabbit anti-TNF-α (1:1,000, #bs-0078R, BIOSS, China), rabbit anti-IL-10 (1:1,000, #bs-20373R, BIOSS, China), mouse anti-glyceraldehyde-3-phosphate dehydrogenase (GAPDH) (1:8,000, #60004-1-Ig, Proteintech Group, United States), and rabbit anti-Tubulin (1:1,000, #11224-1-AP, Proteintech Group, United States) at 4°C overnight. After that, the protein strips were incubated with horseradish peroxidase (HRP)-conjugated secondary antibodies at room temperature for 1h, and analyzed with the Bio-Rad ChemiDoc Imaging System. Bands densities were digitally quantified by Image J software.

### Nissl Staining

The hippocampal tissue sections were mounted and were dehydrated in ascending series of ethanol. Then, the slices were stained with Nissl Staining Solution (Beyotime, China). Finally, the slices were observed under a microscope. At least three sections were taken from each brain. And, all assessments of histological sections were performed blindly.

### Immunohistochemistry and Immunofluorecent

After blocking with QuickBlock Blocking Buffer for Immunol Staining (Beyotime, China), the slices of cells or tissue were incubated with the corresponding primary antibody for goat anti-Iba1 (1:200, #ab5076, Abcam, United Kingdom), mouse anti-iNOS (1:200, # sc-7271, Santa Cruz Biotechnology, United States), or rabbit anti-Arg-1 (1:100, #16001-1-AP, Proteintech Group, United States) overnight at 4°C, and then were incubated with the second antibody (1:500, AlexaFluor-594 and/or 1:500, AlexaFluor-488, Multisciences, China) at 37°C for 1 h. After three washes with phosphate-buffered saline (PBS) for 5 min each, 2-(4-Amidinophenyl)-6-indolecarbamidine dihydrochloride (DAPI) was added to stain nuclei for 5 min. And images were scanned under a confocal laser-scanning microscope (SP8; Leica). Cell numbers were calculated by counting per random microscopic field *via* a blind method. The data are expressed as the number of Iba1^+^ cells per field or the percentage of iNOS^+^ or Arg-1^+^ cells in Iba1^+^ cells. Cell fluorescent signal intensity was quantified using Image J.

### Cell Culture and Model of Inflammation *in vitro*

BV2 cells were purchased from American Type Culture Collection (Manassas, VA, ATCC) and were cultured in Dulbecco’s modified eagle’s medium (DMEM) containing 10% fetal bovine serum (FBS) at 37°C in a humidified incubator with 5% CO_2_. The cells were treated with H_2_S donor (100 μM) for 12 h before being treated with 100 ng/ml lipopolysaccharide (LPS, Escherichia coli serotype 055:B5, sigma, United States) for another 12 h (Yang et al., 2017).

### Statistical Analysis

These results were obtained through more than three independent repeated experiments. Data were analyzed using statistical product and service solutions (SPSS) 25.0 software (SPSS Inc., Chicago, IL, United States) and one-way or two-way ANOVAs, followed by Bonferroni’s *post hoc* test. All data were expressed as the mean ± SEM, and the statistical significance level was set at *p* < 0.05.

## Results

### The H_2_S Donor Reduced Seizures in Pilocarpine-Induced Status Epilepticus Mice

**FIGURE 1:**
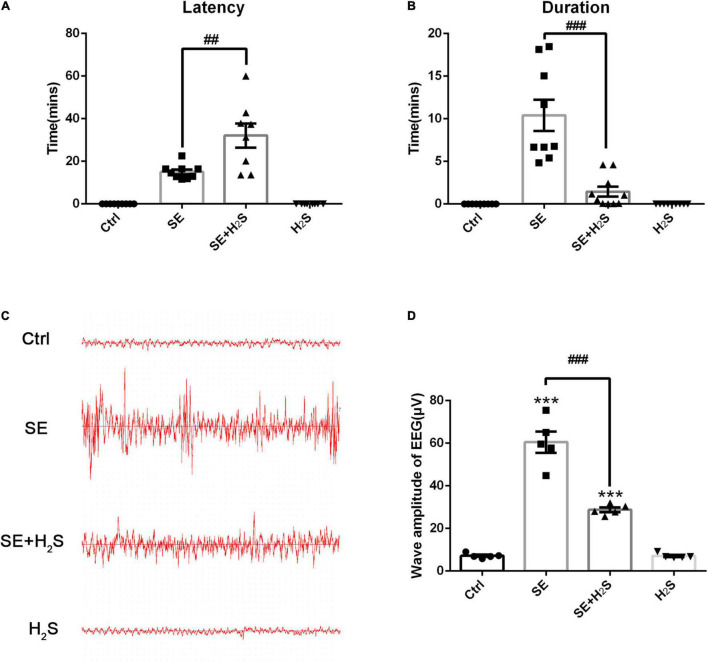
The hydrogen sulfide (H_2_S) donor reduced seizure in a pilocarpine-induced mice model. **(A)** Status epilepticus (SE) mice treated with the H_2_S donor displayed a longer seizure latency. *n* = 9, 9, 8, and 9 for control, SE, SE+H_2_S, and H_2_S groups respectively. **(B)** The H_2_S donor shortened the duration of seizures in SE mice. *n* = 9, 9, 10, and 9 for control, SE, SE+H_2_S, and H_2_S groups respectively. **(C)** Representative EEG waves in mice of each group. **(D)** Statistics data of wave amplitude of electroencephalography (EEG) in different groups. SE mice showed increased wave amplitude of EEG, which was significantly reduced by the H_2_S donor administration. *n* = 5 per group. The results are expressed as the mean ± SEM. ***p < 0.001 vs Control, ^##^*p* < 0.01, ^###^*p* < 0.001 vs SE. One-way ANOVA with Bonferroni *post hoc* tests.

To investigate the effect of the novel H_2_S donor on seizures, we performed i.c.v injection of the H_2_S donor (500 μM, 5 μl) in pilocarpine-induced SE mice. First, the severity of seizures was observed by testing Racine scale. As shown in Figures 1A,B, the control group mice did not appear epileptic seizure. The SE mice treated with the H_2_S donor displayed a longer latency of seizure onset (ANOVA, *p* = 0.001) and a shorter seizure duration (ANOVA, *p* < 0.001) than pilocarpine-induced SE mice. Meanwhile, EEG was applied to record the brain waves of the hippocampus of mice. As shown in Figure 1C, no abnormal discharge was observed in mice of the control group. EEG traces in SE mice showed epileptic brain waves characterized by sharp, spiking, or spiking/slow waves. Consistent with the behavioral observation, administration of the H_2_S donor significantly reduced the epileptic waves (Figure 1C). EEG amplitude analysis in Figure 1D showed that wave amplitudes in SE mice were significantly higher than (ANOVA, *p* < 0.001) that in control mice. And the H_2_S donor decreased the amplitudes of epileptic wave in SE mice (ANOVA, *p* < 0.001) (Figure 1D). These results suggested that the H_2_S donor reduced seizures in pilocarpine-induced mice model.

### The H_2_S Donor Reduced Neuronal Damage in the Hippocampus of Status Epilepticus Mice

**FIGURE 2:**
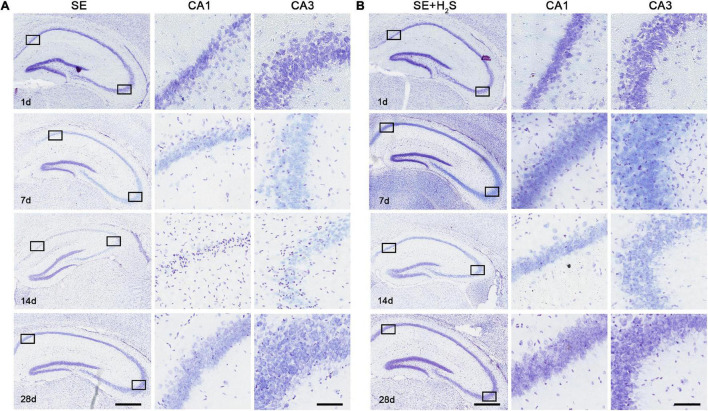
The H_2_S donor reduced neuronal damage in the hippocampus of SE mice. **(A,B)** Nissl staining of hippocampus at different time points (1d, 7d, 14d, 28d) after status epilepticus in SE group and SE+H_2_S group. Scale bars, 25 μm in CA3 and 200 μm in hippocampus. *n* = 5 per group.

Next, we investigated the effect of the novel H_2_S donor on the neuronal damage in different periods after status epilepticus by Nissl staining. At the early stage (1d) after status epilepticus, both CA3 and CA1 areas of the hippocampus showed pyramidal cells arranged densely in line. The Nissl bodies were stained bluish violet and evenly distributed in the cytoplasm, suggesting no obvious morphological damage occurred in the early stage (Figures 2A,B). However, the visible decrease of Nissl bodies occurred at 7d after status epilepticus, reached a peak at 14d, and repaired at 28d. In SE mice, disorder of neuronal arrangement and central chromatolysis were observed in both CA3 and CA1 regions of the hippocampus in progressive stage (7d and 14d). In the convalescent/chronic stage (28d), Nissl body in the cytoplasm partly recovered, and necrosis of neurons were replaced by vacuoles like structures in the tissues (Figures 2A,B). However, SE mice treated with the novel H_2_S donor displayed a better morphology of neurons and more Nissl bodies in cytoplasm in the hippocampus, compared with SE mice. Even on the 14th day of the most severe seizures injury, the complete cell contour was preserved in the H_2_S donor-treated SE mice, but not in SE mice. In convalescent/chronic stage after status epilepticus, there are more dense Nissl bodies and fewer vacuolar structures in the cytoplasm in the hippocampus of the H_2_S donor-treated SE mice. These results suggested that the H_2_S donor decreased the damage of hippocampal neurons in progressive stage of status epilepticus and promoted the repair of neuronal injury in chronic stage.

### The H_2_S Donor Downregulated the Levels of Microglial Pro-inflammatory Profile *in vivo*

**FIGURE 3:**
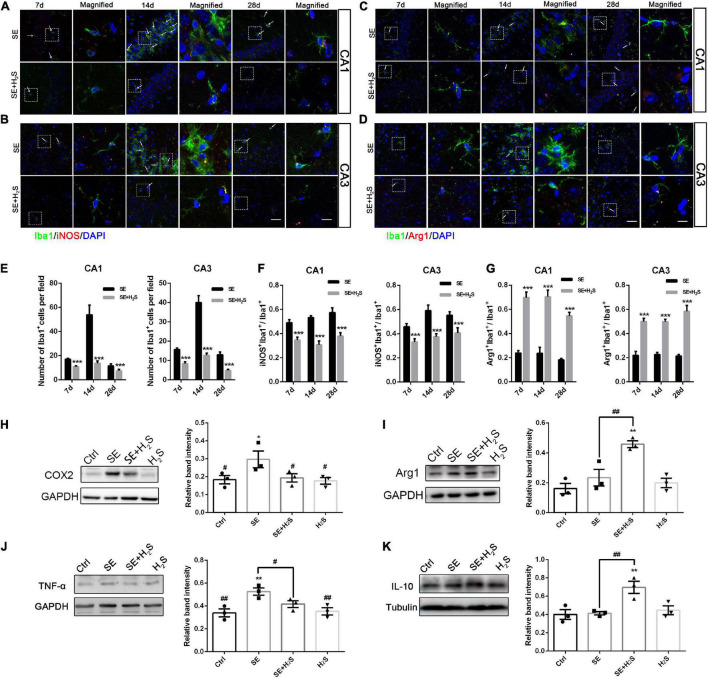
The H_2_S donor downregulated the levels of microglial pro-inflammatory profile *in vivo*. **(A,B)** Representative images of iNOS (red) with Iba1 (green) immunostaining or **(C,D)** Arg1 (red) with Iba1 (green) immunostaining in the CA1 and CA3 regions of the hippocampus in control and the H_2_S donor-treated SE mice. Scale bar 25 μm in the merged panel and 5 μm in the magnified panel. Magnified images are expansions of boxed areas in corresponding panels in the left of each magnified image. **(E)** Quantitative analysis of Iba1^+^cells in CA1 and CA3 regions of the hippocampus. **(F)** Quantitative data are shown the ratio of iNOS^+^: Iba1^+^to total Iba1^+^cells. *n* = 6 per group. ***p < 0.001 *vs* SE. Two-way ANOVA with Bonferroni *post hoc* tests. **(G)** Quantitative data are shown as the ratio of Arg1^+^: Iba1^+^to total Iba1^+^cells. *n* = 6 per group. ***p < 0.001 *vs* SE. Student’s *t* test. **(H–K)** Western blot assay shows that expression levels of microglial pro-inflammatory markers (COX2 and TNF-α) are increased in the hippocampus of SE mice. However, the H_2_S donor decreased COX2 and TNF-α expression and increased Arg-1and IL-10 expression in SE mice. The results are expressed as the mean±SEM. *n* = 3 per group.*p < 0.05, **p < 0.01 *vs* Control,^ #^*p* < 0.05, ^##^*p* < 0.01 *vs* SE. One-way ANOVA with Bonferroni *post hoc* tests.

Microglial activation has been recognized as a major contributor to inflammation of the epileptic brain (Vezzani et al., 2015). We explored the role of the novel H_2_S donor on the inflammatory profile regulation of microglia. A few Iba1^+^cells stained with green were observed in the hippocampus of the control and the H_2_S donor-treated group. These Iba1^+^cells have small cell bodies with a few bifurcations. And there was no significant difference between the two groups in appearance (data not shown). As shown in Figures 3A–D, a number of Iba1^+^cells with different morphologies were observed in the hippocampus of each group. These Iba1^+^cells in SE groups more likely had an enlarged and flat shape cell body with amoeboid appearance. However, in the H_2_S donor-treated SE groups, Iba1^+^cells did not show the inflammatory activation state and showed multi-bifurcated appearance. We quantified the number of Iba1^+^ cells in each group and found that there were more Iba1^+^cells in SE groups [two way-ANOVA, CA1: *F*_(1, 36)_ = 17.37, *p* < 0.001; CA3: *F*_(1, 36)_ = 44.79, *p* < 0.001] (Figure 3E). The number of Iba1^+^cells in both the CA1 and CA3 areas of the hippocampus increased to a peak at 14 d after status epilepticus [two way-ANOVA, CA1: *F*_(2, 36)_ = 13.72, *p* < 0.001; CA3: *F*_(2, 36)_ = 25.30, *p* < 0.001] and declined by 28 d [two way-ANOVA, CA1: *F*_(2, 36)_ = 13.72, *p* < 0.001; CA3: *F*_(2, 36)_ = 25.30, *p* < 0.001] after pilocarpine. We also observed iNOS, a pro-inflammatory marker, co-localized with Iba1. The number of iNOS/Iba1 double-labeled cells was significantly more in SE mice than that in the H_2_S donor-treated SE mice [two way-ANOVA, CA1: *F*_(1, 36)_ = 91.38, *p* < 0.001; CA3: *F*_(1, 36)_ = 64.56, *p* < 0.001] (Figure 3F). In contrast, the co-localized cells of the anti-inflammatory marker Arg1/Iba1 were fewer in SE mice than that in the H_2_S donor-treated SE mice [two way-ANOVA, CA1: *F*_(1, 36)_ = 184.28, *p* < 0.001; CA3: *F*_(1, 36)_ = 153.95, *p* < 0.001) (Figure 3G). The Western blot assay showed that the H_2_S donor treatment alone did not increase the expression of inflammatory profile (such as TNF-α, COX2, IL-10, and Arg1) (Figures 3H–K). However, the H_2_S donor treatment in SE mice not only decreased the expression of microglial pro-inflammatory markers (COX2 and TNF-α) in the hippocampus (ANOVA, COX2: *p* = 0.03; TNF-α: *p* = 0.01) (Figures 3H,J), but also increased the levels of microglial anti-inflammatory markers (Arg1 and IL-10) (ANOVA, Arg1: *p* = 0.002; IL-10: *p* = 0.009) (Figures 3I,K). Taken together, our results indicate that the novel H_2_S donor reduced microglial pro-inflammatory profiles and promoted the anti-inflammatory profiles in the pilocarpine-induced SE mice.

### The H_2_S Donor Downregulated the Levels of Microglial Pro-inflammatory Profile *in vitro*

**FIGURE 4:**
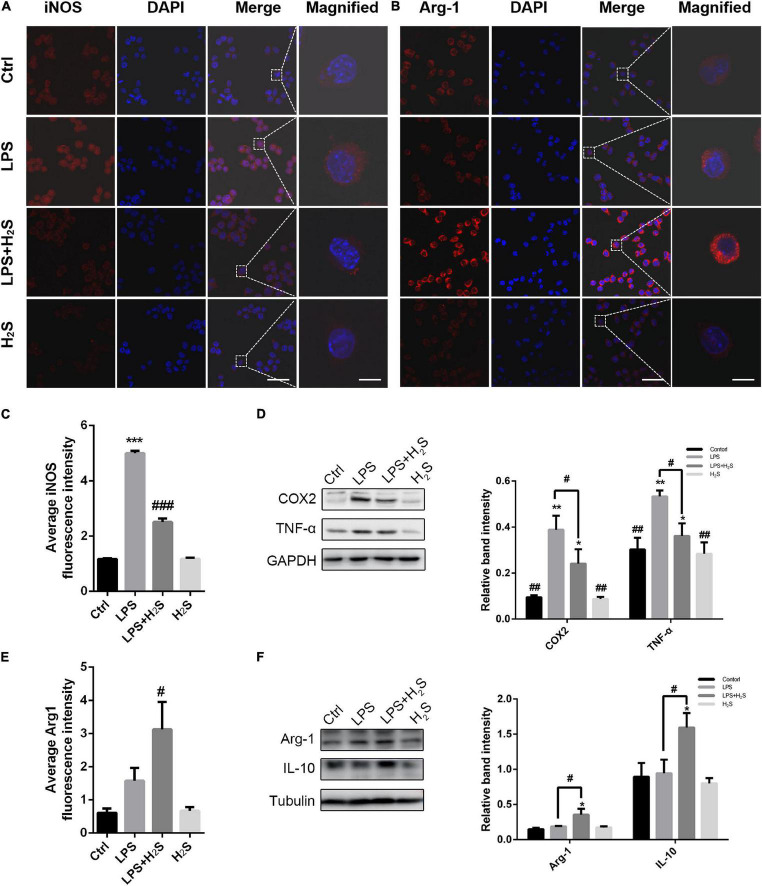
The H_2_S donor downregulated the levels of microglial pro-inflammatory profile *in vitro*. **(A–C,E)** Immunofluorescent staining and quantification of iNOS and Arg1 in lipopolysaccharide (LPS)-treated BV2 cells. Scale bars, 50 μm for the original images and 10 μm for the magnified images. *n* = 6 per group. ***p < 0.001 *vs* Control, ^#^*p* < 0.05, ^###^*p*<0.001 *vs* LPS. Statistical significance was determined by two-way ANOVA with Bonferroni *post hoc* tests. **(D,F)** Expression levels of microglial pro-inflammatory markers (COX2, TNF-α) and microglial anti-inflammatory markers (Arg1, IL-10) in BV2 cells were determined by Western blotting. The H_2_S donor decreased the expression of pro-inflammatory markers and upregulated the expression of anti-inflammatory markers in LPS-induced inflammation model of microglia. *n* = 3 per group. *p < 0.05, **p < 0.01 *vs* Control, ^#^*p* < 0.05, ^##^*p* < 0.01 *vs* LPS. One-way ANOVA with Bonferroni *post hoc* tests.

To further clarify the effect of the H_2_S donor on the inflammatory profile of microglia, we established an inflammation model of microglia induced by LPS in BV2 cells. LPS can result in microglia activation and increase pro-inflammatory cytokines, which is thus known as a representative microglial activation inducer (Yang et al., 2017). As shown in Figures 4A–C,E, LPS significantly increased the pro-inflammatory marker iNOS expression in BV2 cells with no change in the anti-inflammatory marker Arg1 expression. The increased iNOS expression was significantly reduced by administration of the H_2_S donor in LPS-treated BV2 cells (ANOVA, *p* < 0.001). In contrast, the H_2_S donor had an upregulating effect on Arg1 expression (ANOVA, *p* = 0.04). The Western blot assay shows that LPS caused an increase in the expression of the pro-inflammatory markers (COX2 and TNF-α) in BV2 cells (ANOVA, COX2: *p* = 0.001; TNF-α: *p* = 0.008), but not in that of the anti-inflammatory markers (Arg1 and IL10) (Figures 4D,F). Administration of the H_2_S donor significantly reduced the LPS-induced upregulation of the expression of the pro-inflammatory markers (ANOVA, COX2: *p* = 0.004; TNF-α: *p* = 0.02) (Figure 4D), and increased the expression of the anti-inflammatory markers in LPS-treated BV2 cells (ANOVA, Arg1: *p* = 0.03; IL10: *p* = 0.04) (Figure 4F). These results suggested that the H_2_S donor had also regulating effect on inflammatory profile in LPS-induced inflammation model of microglia in BV2 cells.

## Discussion

Hydrogen sulfide has been identified as an important endogenous gasotransmitter regulating various physiological and pathological processes. Recently, we synthesized a novel more stable H_2_S donor, and found it has inhibitory effects on epileptic seizures in a pentetrazol (PTZ)-induced rat model (Zhu et al., 2020, 2021). The present study indicated that the novel H_2_S donor also reduced seizures in pilocarpine-induced mice model. The novel H_2_S donor could prolong the latency to seizure onset, and shorten the duration of seizures. In addition, the H_2_S donor could downregulate the levels of microglial pro-inflammatory profile and increase the levels of microglial anti-inflammatory profile *in vivo* and *in vitro*.

Inflammatory cytokines play an important role in epileptic seizure (Webster et al., 2017; Wang and Chen, 2018). The levels of several pro-inflammatory cytokines such as IL-1β, IL-6, and TNF-α are often elevated in cerebrospinal fluid and serum of patients or rats with epilepsy (Uludag et al., 2013; Webster et al., 2017). These cytokines can increase the excitability of neurons and damage neurons, and are thus thought to be involved in epileptogenesis (Vezzani et al., 2011, 2013). Therefore, anti-inflammatory therapy can effectively reduce the occurrence of epilepsy and chronic seizures. Anti-inflammatory cytokines such as IL-10 may potentially limit epileptogenesis. Both *in vitro* and *in vivo* studies show that H_2_S has regulating effects on various inflammatory factors (Li L. et al., 2009; Huang et al., 2016; Castelblanco et al., 2018). For example, the H_2_S donor (NaHS) administration reduced the expression of microglial pro-inflammatory markers (IL-1β and TNF-α) and concomitantly increased the expression of microglial anti-inflammatory profile (IL-4 and TGF-β) in the brain regions of LPS-treated animals (Kumar et al., 2021). However, the H_2_S administration alone had no effect on basal inflammatory markers (such as COX2, TNF-α, IL-10, and IL-4) (Du et al., 2014; Kumar et al., 2021). Consistent with the present results, the H_2_S donor by itself does not decrease the basal level of TNF-α or IL-10. Thus, the H_2_S only has effects on the changes of inflammatory factors in the disease states, and does not change their basic levels. Besides, the present study also showed that the novel H_2_S donor pretreatment can not only decrease the levels of TNF-α, but also markedly increase the levels of IL-10 in the hippocampus of SE mice. Consistently, morphological observation showed that the H_2_S donor decreased the damage of hippocampal neurons in progressive stage of status epilepticus and promoted the repair of neuronal injury in chronic stage. EEG recordings showed that the H_2_S donor reduced the hippocampal epileptic waves and EEG amplitude. Obviously, the present results of the three effects of the H_2_S donor on the hippocampus are consistent in SE mice.

The microglia are privileged with phenotypic plasticity and can be stimulated by different stimuli to regulate physiological responses and behavioral results in disease (Santos et al., 2016). It is widely accepted that microglial activation occurs following seizures (Eyo et al., 2017; Feng et al., 2019). The activated microglia play a primary role in the production of cytokines. The expression levels of microglial pro-inflammatory cytokines (TNF-α and IL-1β) and anti-pro-inflammator cytokines (IL-10 and IL-4) increase in brain after status epilepticus (Benson et al., 2015). It is reported that abnormally activated microglia, such as stimulated by LPS or kainic acid (KA), aggravate nervous system injury by secreting a variety of pro-inflammatory factors, including IL-1β, IL-12, and TNF-α (Orihuela et al., 2016; Tang and Le, 2016). However, stimulated by specific drugs or cytokines induce microglia to secrete anti-inflammatory factors or express specific genes, such as IL-10, Arg1, which are involved in promoting nerve repair and neurogenesis (Yang et al., 2017; Zhang et al., 2021). This regulation of microglia has been observed in several brain diseases such as Alzheimer’s Disease (Varnum and Ikezu, 2012; Tang and Le, 2016), ischemia (Frieler et al., 2011; Hu et al., 2012), and sclerosis (Henkel et al., 2009; Mikita et al., 2011; Liao et al., 2012; Vogel et al., 2013; Peferoen et al., 2015). It has been shown that pilocarpine-induced status epilepticus was associated with mixed expression of inflammatory profiles (Benson et al., 2015). The present observation also showed this microglial inflammatory regulation during seizures. The pro-inflammatory markers (iNOS and COX2) were upregulated in pilocarpine-induced SE mice. We found that treatment with the novel H_2_S donor in SE mice decreased the expression of microglial pro-inflammatory markers in the hippocampus. Further, the H_2_S donor increased the levels of microglial anti-inflammatory marker Arg1. In LPS-treated microglia BV2 cells, the expression of pro-inflammatory markers (iNOS and COX2) was significantly increased. The novel H_2_S donor reduced the LPS-induced pro-inflammatory marker expression, while it also promoted the release of anti-inflammatory cytokines, as indicated by the increased expression of anti-inflammatory markers (Arg1 and IL-10). Since epilepsy is an inflammation-related disease, our results *in vitro* supported the conclusion of *in vivo* studies that the novel H_2_S donor might regulate the inflammatory of microglia. In a word, the present results indicated that the novel H_2_S donor not only reduced microglial pro-inflammatory profiles, but also simultaneously increased microglial anti-inflammatory profiles. Nowadays, exogenous H_2_S donors, in a variety of experimental systems, were found to induce the activation of signal transduction effects (such as p38, Akt, Erk, JNK, and Stat3), which in turn, produce different functional responses to the expression of various microglia surface antigens and secreted cytokines and exert anti-inflammatory effects (Lee et al., 2010, 2016; Sulen et al., 2016; Zhang et al., 2017; Cao et al., 2018; Li et al., 2020).

In conclusion, our study demonstrated that the novel H_2_S donor can reduce seizures and regulate microglial inflammatory profile. The novel H_2_S donor decreased the release of several pro-inflammatory cytokines (such as TNF-α), which may result in reduced neuronal damage. On the other hand, the H_2_S donor simultaneously increased the release of anti-inflammatory cytokines (such as IL-10), which may result in neuronal recovery. Collectively, our findings identify the H_2_S donor as a potentially approach for seizure neuroprotection.

## Data Availability Statement

The raw data supporting the conclusions of this article will be made available by the authors, without undue reservation.

## Ethics Statement

The animal study was reviewed and approved by Institutional Animal Care and Use Committee of Guangzhou Medical University.

## Author Contributions

XZ and PX designed and conceptualized the experiments. ZL, ZZ, YaH, QK, FL, WZ, YuH, YL, and BH performed the experiments and analyzed the data. ZL and ZZ wrote the manuscript. XZ, PX, and MM revised the manuscript. All authors have read and approved the final manuscript.

## Conflict of Interest

The authors declare that the research was conducted in the absence of any commercial or financial relationships that could be construed as a potential conflict of interest.

## Publisher’s Note

All claims expressed in this article are solely those of the authors and do not necessarily represent those of their affiliated organizations, or those of the publisher, the editors and the reviewers. Any product that may be evaluated in this article, or claim that may be made by its manufacturer, is not guaranteed or endorsed by the publisher.
